# Sex Differences as Predictors of In-Hospital Outcome in Patients with Acute Pulmonary Embolism

**DOI:** 10.3390/jcm15041576

**Published:** 2026-02-17

**Authors:** Corina Cinezan, Camelia Bianca Rus

**Affiliations:** 1 Department of Medical Disciplines, Faculty of Medicine and Pharmacy, University of Oradea, 410073 Oradea, Romania; rus.cameliabianca@student.uoradea.ro; 2 Clinical County Emergency Hospital Bihor, 410169 Oradea, Romania; 3 Doctoral School of Biological and Biomedical Sciences, University of Oradea, 410087 Oradea, Romania

**Keywords:** pulmonary embolism, sex differences, prognosis, risk stratification, right ventricular dysfunction, in-hospital outcomes

## Abstract

**Background**: Sex-related differences in cardiovascular disease outcomes are well recognized. Their impact on short-term outcomes in acute pulmonary embolism (PE) remains unclear. This study aimed to assess the association between sex and in-hospital outcomes in patients with acute PE. **Methods**: We performed a retrospective observational cohort study including 322 consecutive adult patients with acute PE admitted to a university hospital. Clinical, hemodynamic, laboratory, and imaging data were collected at presentation. The primary outcome was a composite poor outcome defined as intensive care unit (ICU) admission, systemic thrombolysis, or in-hospital mortality. Multivariable logistic regression analysis was used to evaluate whether sex independently predicted adverse outcomes after adjustment for established prognostic factors. **Results**: This study included 322 patients with acute pulmonary embolism (mean age 64.4 ± 13.1 years), of whom 50.0% were women. The composite poor outcome occurred more frequently in women than in men (34.0% vs. 22.7%, *p* = 0.032). Female sex was associated with increased odds of poor outcome in univariate analysis (odds ratio (OR) 1.76; 95% confidence interval (CI) 1.08–2.88). This association remained significant after multivariable adjustment (adjusted OR 1.69; 95% CI 1.02–2.82; *p* = 0.042). No significant sex differences were observed for individual components of the composite endpoint. **Conclusions**: Female sex was independently associated with a higher risk of adverse in-hospital outcomes in acute PE, suggesting that sex-specific factors may influence early prognosis and should be considered in future risk stratification models.

## 1. Introduction

Pulmonary embolism represents one of the most common and potentially life-threatening forms of venous thromboembolism, characterized by the obstruction of the pulmonary arteries by thrombotic material originating most often from the deep veins of the lower limbs. Recent population-based analyses continue to demonstrate a substantial and growing burden of pulmonary embolism worldwide, with persistently high short-term morbidity and mortality, particularly among hospitalized and critically ill patients, despite advances in diagnostic and therapeutic strategies [1,2,3]. Accurate risk stratification is therefore essential to guide clinical decision-making, optimize resource allocation, and improve patient outcomes [2,4].

While several clinical, hemodynamic, and laboratory parameters have been identified as prognostic indicators in acute PE, such as hypotension, right ventricular dysfunction, and elevated cardiac biomarkers, less attention has been paid to potential sex-related differences in disease presentation and outcomes [5]. In cardiovascular medicine, sex-based disparities are well established, with women often demonstrating different symptom profiles, delays in diagnosis, and varying prognostic trajectories compared to men [4,6]. However, the influence of sex on the short-term clinical course of acute PE remains less well defined, with existing literature yielding conflicting results. Some studies have reported higher in-hospital mortality or complication rates among women, while others have suggested similar or even more favorable outcomes compared to men [7].

Several biological and clinical mechanisms may underlie these differences. Hormonal modulation of the coagulation cascade, endothelial function, and fibrinolysis, as well as variations in right ventricular adaptation to acute pressure overload, may contribute to sex-specific differences in PE outcomes [4,7]. Furthermore, differences in comorbidity burden, body composition, and treatment response could influence prognosis [6]. Despite these potential contributors, current risk prediction tools, such as the Pulmonary Embolism Severity Index (PESI) and its simplified version, do not incorporate sex as a prognostic variable [2,3].

Despite increasing recognition of sex-specific differences in cardiovascular disease, sex remains absent from most contemporary PE risk stratification tools. Clarifying whether sex independently influences early outcomes may have implications for clinical decision-making, monitoring intensity, and personalized treatment strategies.

Given these uncertainties, the present study aimed to investigate the relationship between sex and clinical outcomes among patients with acute pulmonary embolism. Specifically, we sought to determine whether female sex was independently associated with a higher risk of adverse in-hospital events, including intensive care unit admission, thrombolytic therapy, or in-hospital mortality. We hypothesized that women with acute PE would experience a higher incidence of poor outcomes compared with men, even after adjustment for established prognostic factors.

## 2. Materials and Methods

Study Design and Setting

This retrospective observational cohort study was conducted at the Cardiology Department of the Clinical County Emergency Hospital Bihor, a tertiary care university-affiliated center providing 24 h emergency cardiovascular care. The hospital serves as a regional referral center for acute pulmonary embolism (PE), including intermediate- and high-risk cases requiring advanced monitoring or reperfusion therapy.

All consecutive adult patients admitted between January 2022 and October 2025 with a diagnosis of acute PE were screened for eligibility.

Study Population

Acute PE was diagnosed based on computed tomography pulmonary angiography (CTPA) demonstrating intraluminal filling defects in segmental or more proximal pulmonary arteries, in accordance with ESC guidelines. Only patients with objectively confirmed acute PE were included.

Inclusion criteria: age ≥ 18 years, confirmed diagnosis of acute PE, and availability of complete clinical, laboratory, imaging, and outcome data.

Exclusion criteria: chronic or subacute PE (>14 days from symptom onset), recurrent PE during the same hospitalization, severe concomitant infection or sepsis, pregnancy, and incomplete or missing key clinical data.

After applying these criteria, 322 patients constituted the final study cohort.

**Figure 1:**
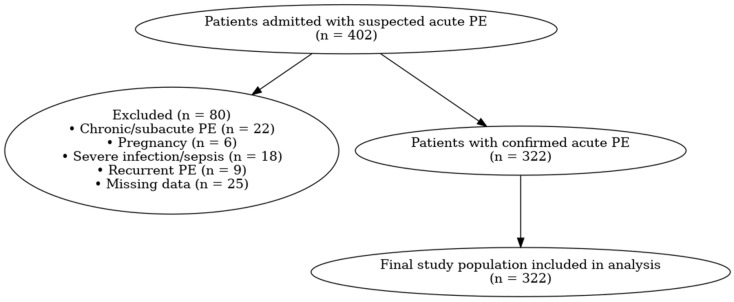
Flow diagram of patient selection for this study.

A flow diagram illustrating patient screening, exclusions, and final study inclusion is presented in Figure 1.

Data Collection

Clinical data were extracted from the electronic medical record system by two investigators using a standardized data collection form. Discrepancies were resolved by consensus.

Collected variables included: demographic data (age, sex), hemodynamic parameters at admission (heart rate, systolic blood pressure), comorbidities (arterial hypertension, diabetes mellitus, cardiovascular disease, active malignancy, prior venous thromboembolism), laboratory biomarkers (D-dimer, cardiac troponin), and imaging findings from echocardiography and CTPA.

Definition of Clinical Variables

Hypotension was defined as systolic blood pressure < 90 mmHg at presentation.

Tachycardia was characterized as heart rate ≥ 100 beats/min

Elevated troponin was defined as the value above the institutional upper reference limit

Right ventricular (RV) dysfunction was defined as any of the following: RV/LV diameter ratio ≥ 1.0 on CTPA or echocardiographic RV dilation or hypokinesia.

All measurements and definitions followed contemporary ESC recommendations.

Outcome Measures

The primary outcome was a composite endpoint termed poor in-hospital outcome, defined as the occurrence of at least one of the following: admission to the intensive care unit (ICU), administration of systemic thrombolytic therapy, and in-hospital mortality.

Secondary outcomes included each component of the composite endpoint analyzed individually.

This study was reported in accordance with the STROBE (Strengthening the Reporting of Observational Studies in Epidemiology) guidelines. The completed STROBE checklist is provided as Supplementary Table S1.

Statistical Analysis

Continuous variables were summarized as mean ± standard deviation (SD) for normally distributed data or median [interquartile range, IQR] for non-normally distributed data. Categorical variables were expressed as counts and percentages. Comparisons between women and men were performed using the Chi-square test or Fisher’s exact test for categorical variables and the independent-samples *t*-test or Mann–Whitney U test for continuous variables, as appropriate. Univariate associations between sex and each clinical outcome were evaluated using odds ratios with 95% confidence intervals. Multivariable logistic regression was used to identify independent predictors of poor outcome, adjusting for potential confounders including age, hypotension, active malignancy, right ventricular dysfunction, tachycardia, and elevated troponin. The model’s discriminative ability was assessed using the area under the receiver operating characteristic curve (AUC). All tests were two-tailed, and a *p*-value < 0.05 was considered statistically significant. Analyses were conducted using SPSS version 29.0 (IBM Corp., Armonk, NY, USA) and R version 4.2.3 (R Foundation for Statistical Computing, Vienna, Austria).

A post-hoc power analysis indicated that, given the observed effect size for the composite outcome (OR 1.76), the available sample size provided an estimated statistical power exceeding 0.80 at a two-sided α level of 0.05, supporting the adequacy of the cohort for detecting clinically meaningful sex-related differences.

## 3. Results

A total of 322 consecutive patients with objectively confirmed pulmonary embolism were analyzed, of whom 161 (50.0%) were women. Baseline demographic and clinical characteristics according to sex are presented in Table 1 and Table 2.

Women and men were similarly distributed in terms of age, hemodynamic parameters, and laboratory findings at presentation (Table 1). No statistically significant sex-related differences were observed for continuous variables, including heart rate, systolic blood pressure, D-dimer levels, right ventricular dimensions, RV/LV ratio, or cardiac troponin concentrations.

Regarding categorical variables, women showed numerically higher rates of active malignancy, arterial hypertension, previous venous thromboembolism, elevated troponin, and right ventricular dysfunction compared with men, although these differences did not reach statistical significance (Table 2). The prevalence of hypotension and tachycardia on admission was similar between sexes.

Women were slightly older on average and exhibited similar prevalence rates of comorbidities such as arterial hypertension, diabetes mellitus, and cardiovascular disease compared with men. No major sex-related differences were observed in baseline hemodynamic parameters or right ventricular function.

**Figure 2:**
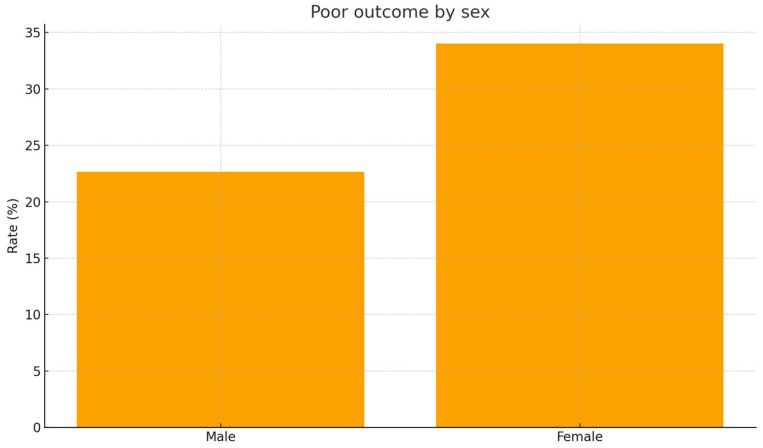
Composite poor outcome by sex. Bar chart illustrating the incidence of composite poor outcome in men versus women. Poor outcome is defined as ICU admission, thrombolysis, or in-hospital mortality. Women experienced significantly higher adverse event rates (34.0% vs. 22.7%, *p* = 0.032).

The overall incidence of the composite poor outcome, defined as intensive care unit admission, administration of thrombolytic therapy or in-hospital mortality, was 27.6%. The poor outcome occurred in 34.0% of women and 22.7% of men (*p* = 0.032), indicating a significantly higher event rate among female patients (Figure 2).

**Figure 3:**
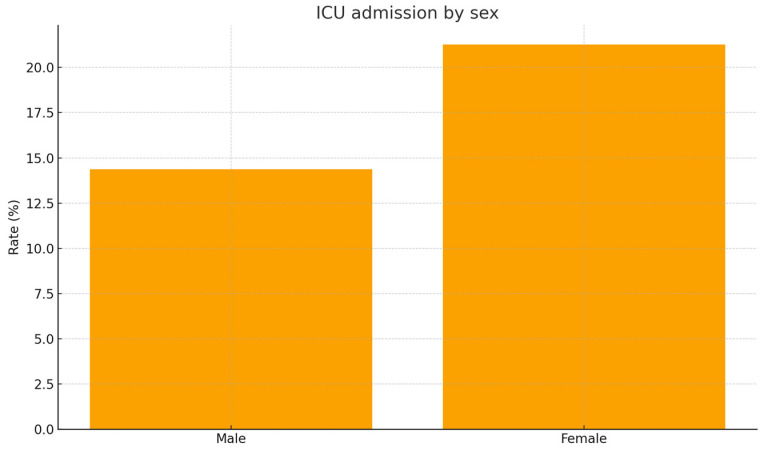
ICU admission by sex. Bar plot comparing the proportion of patients requiring intensive care unit admission according to sex. Women showed a higher numerical rate of ICU admission than men, though without statistical significance (21.3% vs. 14.4%, *p* = 0.14).

When analyzed individually, ICU admission occurred in 19.3% of women versus 13.1% of men (*p* = 0.09; Figure 3).

**Figure 4:**
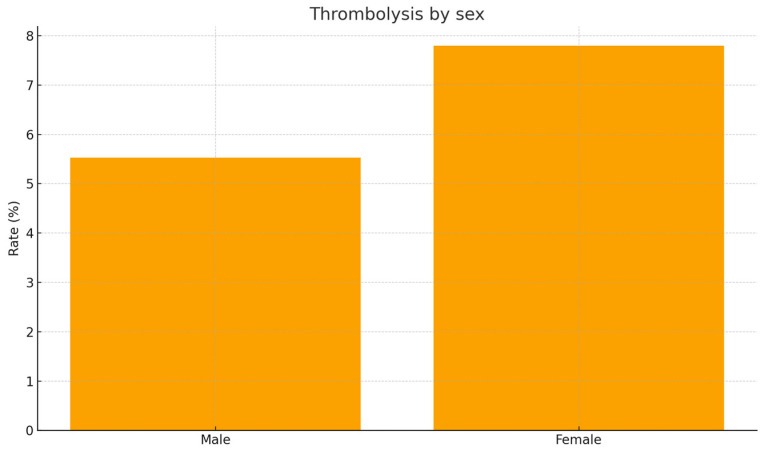
Rate of thrombolytic therapy by sex. Figure displays percentage of patients undergoing systemic thrombolysis in each sex group. Women required thrombolysis more often numerically, but without statistical significance (7.8% vs. 5.5%, *p* = 0.55).

Thrombolytic therapy was required in 6.8% women versus 4.1% men (*p* = 0.28; Figure 4).

**Figure 5:**
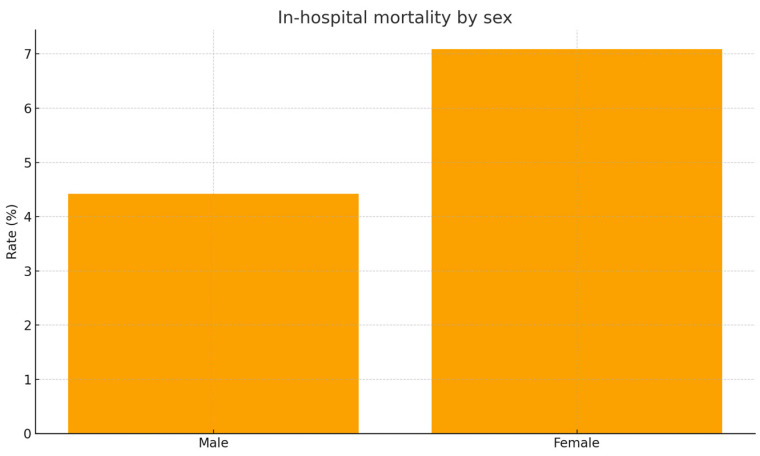
In-hospital mortality by sex. Plot showing sex-stratified mortality among hospitalized pulmonary embolism patients. Women demonstrated slightly higher mortality, though differences were not statistically significant (7.1% vs. 4.4%, *p* = 0.43).

Finally, in-hospital mortality occurred in 8.7% women versus 5.6% men (*p* = 0.26; Figure 5).

While individual outcomes did not differ significantly between sexes, the cumulative burden of adverse events was significantly higher in women, indicating that sex-related differences may emerge only when outcomes are considered jointly rather than in isolation. None of these differences reached statistical significance. These findings suggest that the observed sex difference was driven by the cumulative occurrence of adverse events rather than by a single dominant outcome.

Univariate analyses of all outcome variables are summarized in Table 3.

In univariate analyses, female sex was significantly associated with an increased risk of the composite poor outcome (OR 1.76; 95% CI 1.08–2.88; *p* = 0.032). In contrast, female sex was not significantly associated with ICU admission, thrombolysis, or in-hospital mortality when these outcomes were analyzed individually.

In unadjusted analyses, female sex was associated with increased odds of poor outcome (OR 1.76; 95% CI 1.08–2.88; *p* = 0.032). After adjustment for age, hypotension (systolic blood pressure < 90 mmHg), active malignancy, right ventricular dysfunction, tachycardia, and elevated troponin in the multivariable logistic regression model, female sex remained an independent predictor of poor outcome (adjusted OR 1.69; 95% CI 1.02–2.82; *p* = 0.042). Multivariable logistic regression results, including Wald χ^2^ statistics, are presented in Table 4.

Female sex remained an independent predictor of poor outcome (OR 1.69, *p* = 0.042), RV dysfunction trended toward significance and may represent an intermediate-risk signaling marker, and troponin and hypotension did not retain predictive significance after adjustment.

The persistence of female sex as an independent predictor after multivariable adjustment suggests that unmeasured biological or clinical factors may contribute to early in-hospital vulnerability beyond established prognostic markers.

Among other covariates, right ventricular dysfunction demonstrated a trend toward association with adverse outcomes, while elevated troponin and hypotension were not significant predictors in the adjusted model.

In summary, women with acute pulmonary embolism exhibited a higher risk of composite poor outcomes during hospitalization compared with men, a difference that persisted after adjustment for relevant clinical confounders. No significant sex-related differences were observed for individual components of the composite endpoint—ICU admission, thrombolysis, or in-hospital mortality—when analyzed separately.

## 4. Discussion

In this study of 322 patients with objectively confirmed pulmonary embolism (PE), we observed that women had a significantly higher incidence of composite poor outcomes compared with men, a finding that persisted after adjustment for relevant clinical covariates. The poor outcome, defined as ICU admission, thrombolysis, or in-hospital mortality, occurred in 34% of women versus 23% of men, and female sex remained an independent predictor of adverse in-hospital events in the multivariable model. These results highlight potential sex-related differences in the clinical presentation, pathophysiologic response, and management of acute PE.

Several contemporary registries and meta-analyses have reported sex-related differences in PE presentation, management, and outcomes, although results remain heterogeneous, underscoring the need for further targeted investigation.

Our findings align with several prior studies suggesting that women may experience worse short-term outcomes following acute PE [8,9]. Possible explanations include biological differences in right ventricular adaptation to pressure overload, hormonal influences on vascular reactivity and coagulation, and differences in thrombotic burden or clot distribution [10]. Estrogen-related modulation of the fibrinolytic system and endothelial function has been proposed as a potential mechanism contributing to more severe presentations or slower hemodynamic recovery in female patients. In addition, postmenopausal status and associated comorbidities may exacerbate right ventricular strain and limit compensation during acute PE [7].

From a clinical perspective, the observed sex difference may also reflect variations in management strategies, diagnostic timing, or treatment thresholds. Prior research has reported that women are sometimes less likely to receive early reperfusion therapy or invasive hemodynamic monitoring, potentially due to underestimation of disease severity [9,11]. Although our analysis did not identify a significant sex disparity in the use of thrombolysis or ICU admission, these subtle differences in treatment approach could contribute to the higher overall rate of adverse outcomes among women [12]. It is also possible that differences in body composition or pharmacokinetics influence the efficacy and safety of anticoagulation therapy [13].

Interestingly, while female sex was associated with the composite poor outcome, the association was not significant for individual components, such as in-hospital mortality or thrombolysis, when analyzed separately [10,14]. This pattern suggests that the increased risk in women may derive from the cumulative burden of moderate clinical complications rather than from isolated fatal events [9]. This observation is consistent with previous registry data showing comparable mortality rates between sexes but higher resource utilization and longer hospital stays among women [8,15].

Our study contributes to the growing body of evidence emphasizing the need for sex-specific risk assessment in acute PE [16]. Traditional risk models, such as the Pulmonary Embolism Severity Index (PESI) or simplified PESI, may not fully capture the interaction between sex and physiologic response. Incorporating sex as a variable in future prognostic models could improve risk stratification and optimize treatment allocation [17,18]. Furthermore, mechanistic research investigating sex-related differences in right ventricular adaptation, microvascular function, and hormonal modulation of thrombus formation and inflammation may provide insight into the observed disparities [19].

This study has several strengths. First, it included a well-defined cohort of consecutive patients with objectively confirmed acute PE, ensuring diagnostic accuracy and minimizing selection bias. Second, comprehensive clinical, hemodynamic, and laboratory data were available for all participants, allowing for robust adjustment for potential confounders, including right ventricular dysfunction, troponin elevation, and systemic hypotension. Third, the outcomes were clearly defined and clinically meaningful, capturing both severe and intermediate-risk complications through a composite endpoint. Fourth, the use of a multivariable logistic regression framework strengthened the validity of the associations by accounting for multiple concurrent predictors. Finally, the dedicated focus on sex-related outcomes in PE provides valuable insights into an area that remains relatively underexplored in the current literature. The balanced sex distribution in this cohort further enhances the generalizability of the findings to real-world populations.

Nevertheless, several important limitations must be acknowledged. First, the retrospective and single-center design limits causal inference and introduces potential selection bias. The single-center design of this study may limit the generalizability of the findings, as patient characteristics, referral patterns, and management strategies can vary across institutions, potentially influencing observed outcomes [20]. Although consecutive patients were included, referral and admission patterns may have influenced the study population, potentially limiting the external validity of our findings. Second, as with most retrospective analyses, unmeasured confounding cannot be excluded. Factors such as hormonal status, menopausal stage, body mass index, or medication use (hormone replacement therapy or oral contraceptives) were not systematically recorded and could partly explain the observed sex differences. Third, imaging assessments of right ventricular dysfunction were not standardized across all cases and may have varied depending on operator expertise and modality (echocardiography versus CT). Fourth, the sample size, although moderate, may not have provided adequate power for detecting sex differences in less frequent outcomes such as thrombolysis or mortality. Fifth, our study was limited to in-hospital outcomes; therefore, longer-term prognostic implications and functional recovery differences between sexes remain unknown. Sixth, laboratory assays for biomarkers such as troponin and D-dimer were not harmonized across time, which may introduce minor variability in the classification of elevated values.

Finally, the cohort was derived from a single tertiary hospital, which may limit generalizability to other settings with differing management practices, case mix, or healthcare resources. Despite these limitations, this study’s comprehensive data collection, objective diagnostic confirmation, rigorous definition of endpoints, and multivariable modeling provide a robust framework for interpreting the observed sex-related differences in outcomes.

Future Directions

The results of this study underscore the importance of considering sex as an independent factor in the prognostic assessment and clinical management of acute pulmonary embolism. Future research should focus on validating these findings in larger, multicenter cohorts and diverse populations to confirm the consistency of sex-related differences in short-term and long-term outcomes. Prospective studies incorporating detailed hormonal profiles, menopausal status, and sex-specific biomarkers may help clarify the underlying mechanisms driving the observed disparities. Furthermore, integrating sex-based parameters into existing prognostic tools such as the PESI or simplified PESI may improve their predictive accuracy and enhance individualized risk stratification. Our findings may inform future updates of prognostic models and encourage heightened surveillance strategies for women hospitalized with acute pulmonary embolism.

Beyond prognostic modeling, future investigations should also address potential differences in treatment response and post-discharge recovery between men and women. This includes examining sex-specific thresholds for thrombolytic therapy, optimal anticoagulant dosing, and long-term outcomes such as recurrent venous thromboembolism, chronic thromboembolic pulmonary hypertension, and functional status. A more nuanced understanding of these factors will inform precision medicine approaches and support the development of tailored clinical pathways that account for biological and clinical sex-related variations in PE outcomes.

## 5. Conclusions

Female sex was independently associated with a higher risk of adverse in-hospital outcomes among patients with acute pulmonary embolism. The observed differences likely reflect a complex interplay of biological, physiological and clinical factors. These findings underscore the importance of recognizing sex as a potential prognostic determinant in PE and warrant further investigation in prospective, multicenter studies to confirm and clarify the mechanisms driving this disparity.

## Figures and Tables

**Table 1:** Baseline characteristics by sex, with continuous variables summarized as mean ± SD and median [IQR].

Variable	N—Female	N—Male	Mean ± SD—Female	Mean ± SD—Male	Median [IQR]—Female	Median [IQR]—Male
Age (years)	161	161	63.0 ± 13.4	65.8 ± 12.8	65.0 [53.0–73.0]	66.0 [57.0–74.0]
D-dimer (ng_mL)	161	161	2315.9 ± 1175.8	2522.6 ± 1230.3	2241.0 [1513.0–2913.0]	2278.0 [1547.0–3372.0]
Heart rate (bpm)	161	161	97.4 ± 20.4	99.6 ± 19.5	99.0 [85.0–113.0]	100.0 [86.0–113.0]
RV basal diameter (mm)	161	161	38.7 ± 6.6	37.3 ± 6.6	38.1 [34.4–42.4]	36.6 [32.8–40.7]
RV/LV ratio	161	161	1.0 ± 0.2	1.0 ± 0.2	1.0 [0.9–1.1]	0.9 [0.8–1.0]
Sbp (mmHg)	161	161	119.5 ± 17.3	117.6 ± 16.7	121.0 [109.0–132.0]	118.0 [107.0–129.0]
Troponin (ng/L)	161	161	92.4 ± 152.7	80.1 ± 164.3	14.0 [8.2–133.5]	9.9 [7.3–99.8]

Notes**:** RV/LV ratio represents right ventricular size relative to the left ventricle on imaging assessment, with troponin levels reported in ng/L using institutional thresholds for elevation, systolic blood pressure (SBP) measured at presentation in the emergency department and no statistically significant sex-related differences observed for any continuous variable. N reflects the number of patients with complete data available for each continuous variable.

**Table 2:** Baseline characteristics by sex, withcategorical variables summarized as counts (%).

Variable	Female	Male
Active malignancy	22 (15.6%)	18 (9.9%)
Arterial hypertension	60 (42.6%)	59 (32.6%)
Cardiovascular disease	32 (22.7%)	25 (13.8%)
Diabetes mellitus	26 (18.4%)	30 (16.6%)
Elevated troponin	65 (46.1%)	56 (30.9%)
Previous VTE	20 (14.2%)	16 (8.8%)
RV dysfunction	47 (33.3%)	40 (22.1%)
Sbp < 90 mmHg	10 (7.1%)	10 (5.5%)
Syncope	0 (0.0%)	53 (29.3%)
Tachycardia	68 (48.2%)	95 (52.5%)

Notes**:** RV dysfunction is defined as RV/LV ≥ 1.0 on CT or echo or qualitative hypokinesia or tachycardia = HR ≥ 100 bpm. SBP < 90 mmHg indicates hemodynamic instability upon presentation, with elevated troponin classified per hospital reference thresholds. Women demonstrated numerically higher rates of active malignancy, hypertension and troponin elevation.

**Table 3:** Univariate associations between sex and clinical outcomes.

Outcome	Women n/N (%)	Men n/N (%)	OR Female vs. Male (95% CI)	*p*-Value
Poor outcome	48/141 (34.0%)	41/181 (22.7%)	1.76 (1.08–2.88)	0.032
ICU admission	30/141 (21.3%)	26/181 (14.4%)	1.61 (0.90–2.87)	0.1401
Thrombolysis	11/141 (7.8%)	10/181 (5.5%)	1.45 (0.60–3.51)	0.5529
In-hospital mortality	10/141 (7.1%)	8/181 (4.4%)	1.65 (0.63–4.30)	0.4289

Notes: Composite poor outcome = ICU admission OR thrombolysis OR in-hospital mortality, with female sex associated with poorer outcomes in unadjusted analysis (*p* = 0.032). Individual endpoints did not reach significance, suggesting cumulative rather than isolated event-driven risk. Categorical variables were compared using the chi-square test. Odds ratios were derived from univariate logistic regression. Effect size is expressed as odds ratio (OR) with 95% confidence interval (CI), alongside *p*-values and statistical tests.

**Table 4:** Multivariable logistic regression analysis for composite poor outcome.

Predictor	OR (95% CI)	Wald χ^2^	*p*-Value
Female sex	1.69 (1.02–2.82)	4.12	0.042
Age (years)	0.99 (0.97–1.01)	0.80	0.371
SBP < 90 mmHg	1.52 (0.57–4.05)	0.69	0.405
Active malignancy	1.02 (0.47–2.22)	0.00	0.958
RV dysfunction	1.60 (0.93–2.75)	2.92	0.088
Elevated troponin	0.79 (0.46–1.34)	0.77	0.384
Tachycardia	1.10 (0.67–1.82)	0.14	0.708

Notes**:** Adjusted regression model evaluating female sex alongside clinical covariates for prediction of composite poor outcome. OR > 1 indicates greater risk contribution. Final model adjusted for age, hypotension, active malignancy, RV dysfunction, elevated troponin, and tachycardia.

## Data Availability

The raw data supporting the conclusions of this article will be made available by the authors upon request. The original contributions presented in this study are included in the article. Further inquiries can be directed to the corresponding author.

## Supplementary Materials

The following supporting information can be downloaded at https://www.mdpi.com/article/10.3390/jcm15041576/s1. Supplementary Table S1. STROBE Checklist, STROBE (Strengthening the Reporting of Observational Studies in Epidemiology) checklist for the study entitled “Sex Differences as Predictors of In-Hospital Outcome in Patients with Acute Pulmonary Embolism”.
