# Supplementary material for: Sex Differences as Predictors of In-Hospital Outcome in Patients with Acute Pulmonary Embolism
Source: J Clin Med. 2026 Feb 17;15(4):1576. doi: 10.3390/jcm15041576 (PMC12941874; doi:10.3390/jcm15041576)
Supplement: Supplementary file 1 [file jcm-15-01576-s001.zip › jcm-4141255-supplementary.pdf]

Supplementary Table S1. STROBE Checklist

STROBE (Strengthening the Reporting of Observational Studies in Epidemiology) checklist for the study entitled “Sex Differences as Predictors of In-Hospital Outcome in Patients with Acute Pulmonary Embolism.”

Section	Item No.	Recommendation	Page/Location in Manuscript
Title/Abstract	1	Indicate the study design with a commonly used term	Title, Abstract
Introduction	2	Explain the scientific background and rationale	Introduction
Introduction	3	State specific objectives and hypotheses	End of Introduction
Methods	4	Present key elements of study design	Materials and Methods
Methods	5	Describe the setting, locations, and relevant dates	Study Design and Population
Methods	6	Give eligibility criteria and selection methods	Inclusion and Exclusion Criteria
Methods	7	Clearly define outcomes, exposures, predictors	Data Collection and Outcomes
Methods	8	Describe data sources and measurement	Data Collection
Methods	9	Describe efforts to address potential sources of bias	Limitations
Methods	10	Explain how the study size was determined	Study Population
Methods	11	Explain handling of quantitative	Statistical Analysis

		variables	
Methods	12	Describe all statistical methods	Statistical Analysis
Results	13	Report numbers of individuals at each stage of the study	Figure 1
Results	14	Give characteristics of study participants	Tables 1 and 2
Results	15	Report outcome data	Tables 3 and 4
Discussion	16	Summarize key results with reference to study objectives	Discussion
Discussion	17	Discuss limitations of the study	Limitations subsection
Discussion	18	Give a cautious interpretation of results	Discussion
Other	19	Discuss generalizability of the results	Discussion
Other	20	Give the source of funding and role of funders	Funding Statement
